# Supplementary material for: DMRforPairs: identifying Differentially Methylated Regions between unique samples using array based methylation profiles
Source: BMC Bioinformatics. 2014 May 15;15:141. doi: 10.1186/1471-2105-15-141 (PMC4046028; doi:10.1186/1471-2105-15-141)
Supplement: Additional file 2 — DMRforPairs output for the comparison of A431-MCF7 and NA17018-NA17105. Please start from the HTML files in each folder. Available via the BMC Bioinformatics website. [file 1471-2105-15-141-S2.zip › 1394847754114233_MOESM2_ESM/A431_MCF7/figures/26215.pdf]

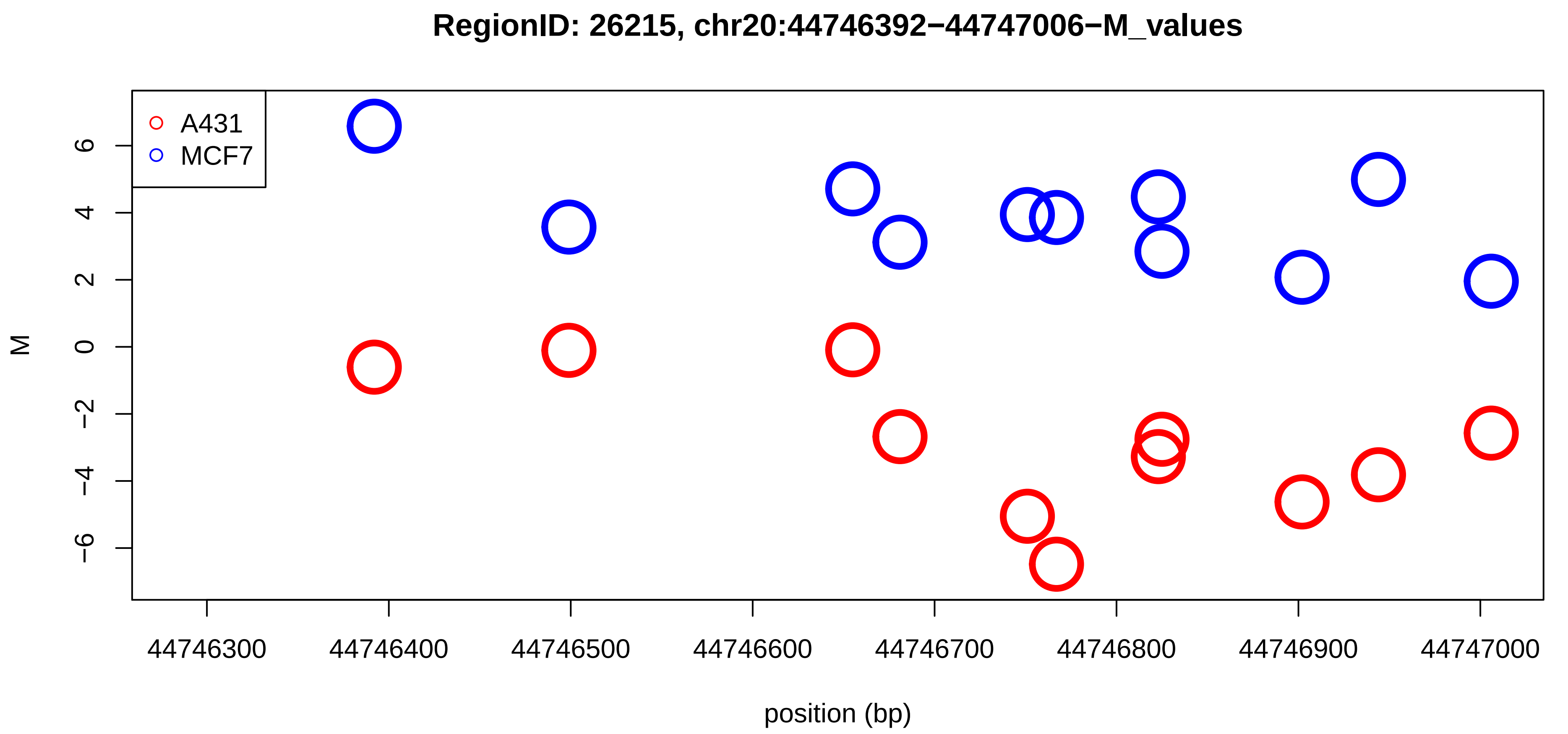
RegionID: 26215, chr20:44746392−44747006−M_values
A431
MCF7
M
position (bp)

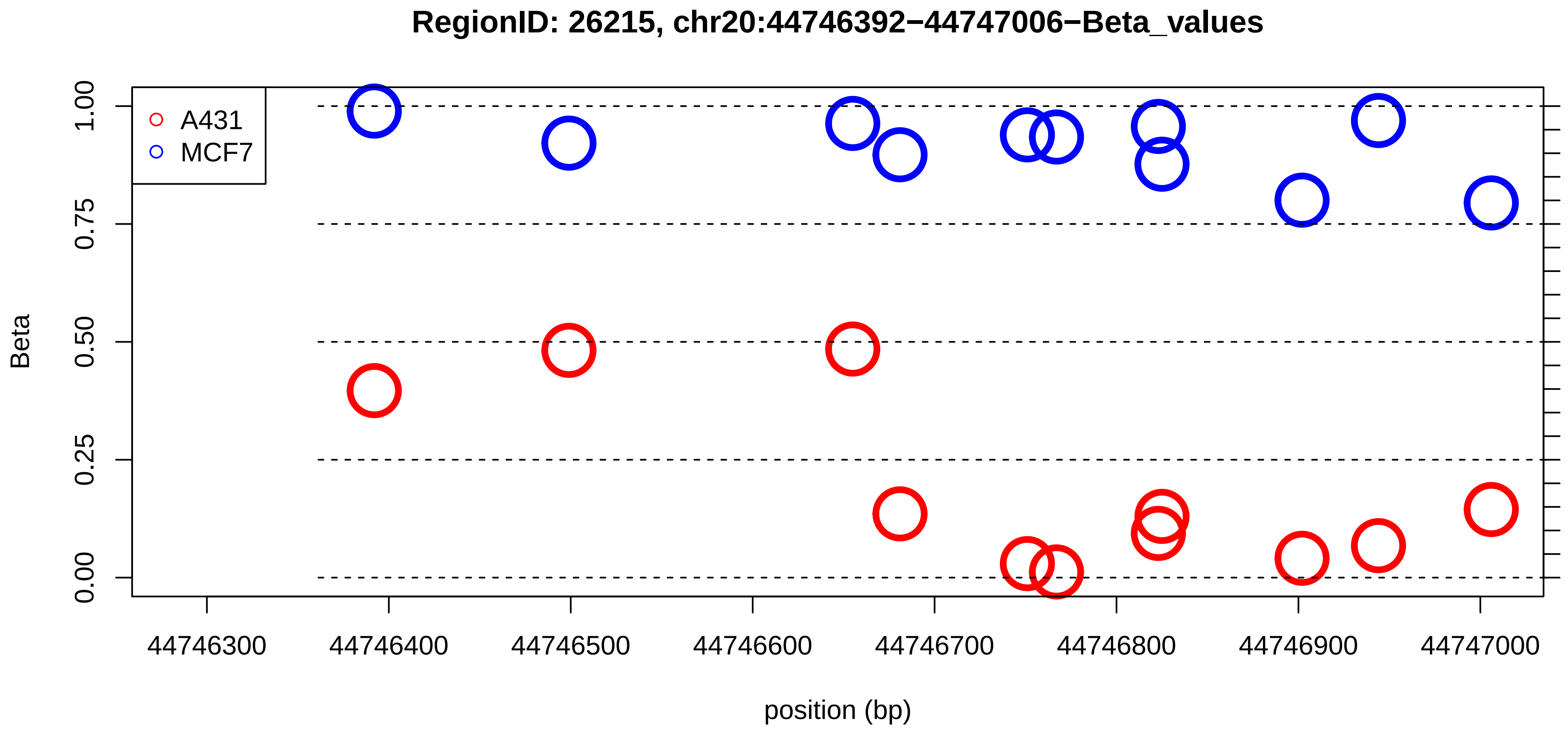
RegionID: 26215, chr20:44746392−44747006−Beta_values
A431
MCF7
Beta
position (bp)

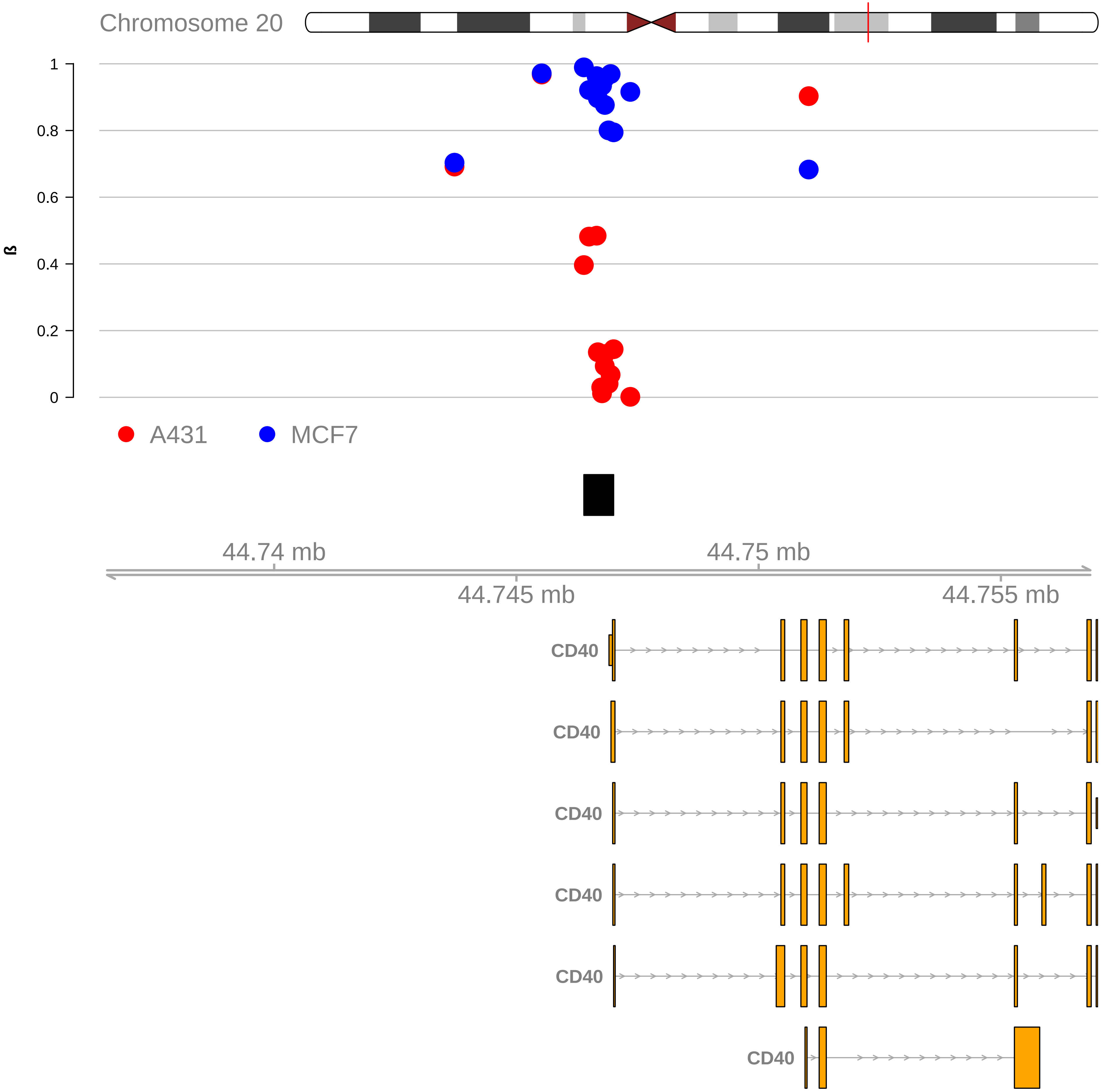
Chromosome 20
1
0.8
0.6
0.4
0.2
0
ß
A431
MCF7
44.74 mb
44.75 mb
44.745 mb
44.755 mb
CD40
CD40
CD40
CD40
CD40
CD40
